# Probiotics in type 1 diabetes: immunomodulatory mechanisms and the unresolved question of clinical efficacy

**DOI:** 10.3389/fimmu.2026.1931902

**Published:** 2026-10-07

**Authors:** Simiao Chen, Lu Wang, Shuotong Zhang, Jun Zhang, Xin Yan, Zhongming Wu

**Affiliations:** 1 Key Laboratory of Endocrine Glucose & Lipids Metabolism and Brain Aging, Ministry of Education, Department of Endocrinology, Shandong Provincial Hospital Affiliated to Shandong First Medical University, Jinan, Shandong, China; 2 Department of Oncology, Provincial Hospital Affiliated to Shandong First Medical University, Jinan, Shandong, China

**Keywords:** probiotics, type 1 diabetes, immunity, delivery technology, personalized medicine

## Abstract

Type 1 Diabetes (T1D) is a common autoimmune disease worldwide, which is associated with imbalanced gut microbiota. Intestinal dysbiosis plays a key role in the pathogenesis of T1D, and it may affect immune tolerance and autoimmune responses of pancreatic islet β-cells. As a potential immunomodulator, probiotics can help restore this balance, regulate immune responses, and reduce the risk of T1D. Although probiotics offer a logical immunomodulatory strategy to restore microbial and immune homeostasis, the translational gap between robust preclinical mechanisms and inconsistent clinical outcomes remains poorly defined. This review summarizes the mechanisms of action and clinical research progress of probiotics in the immunotherapy of T1D. Firstly, it elaborates on the key pathways through which probiotics regulate the gut-immune-pancreas axis. Subsequently, it conducts a comparative analysis of important clinical trials on probiotic therapy for T1D in recent years, comparing the differences in efficacy of different intervention timings, different bacterial strains, etc., in the prevention and adjuvant treatment of T1D, and explores their relationships with patients’ age, disease duration, and genetic background. Meanwhile, it points out the limitations of current research, so as to provide important references for future studies. Furthermore, this review looks forward to the future directions of probiotics in the field of T1D immunotherapy, including innovative approaches such as engineered bacteria transformation, targeted delivery, and personalized microbiota intervention. Finally, it evaluates the safety of probiotics in the treatment of T1D, aiming to explore an individualized probiotic therapy method and lay a more reliable foundation for subsequent clinical translation.

## 1 Introduction

Type 1 diabetes (T1D) is a complex autoimmune disease mediated by autoreactive T lymphocytes, which is associated with immune dysfunction of pancreatic islet β-cells (1). Its incidence rate has been on the rise globally. As of 2024, the global prevalence of T1D has reached 9.15 million cases, with 503,000 new cases diagnosed annually (2). It is projected that the number of prevalent cases may increase to 13.5 million to 17.4 million by 2040 (3).

Currently, there are multiple treatment approaches for T1D. The core therapy is exogenous insulin replacement, combined with continuous glucose monitoring to optimize glycemic control (4, 5). In addition, there are disease-modifying therapies (e.g., Teplizumab) to delay disease progression, as well as approaches such as stem cell therapy and pancreatic transplantation (6–8). However, these methods have obvious limitations: insulin therapy cannot perfectly mimic physiological insulin secretion, and is associated with risks including hypoglycemia and low compliance (9). Moreover, some automated insulin delivery (AID) systems are not yet suitable for patients with young ages or low-dose requirements (10). Immunomodulatory therapies offer only transient efficacy, require sustained administration, and fail to provide clinical benefits in all individuals (11, 12). At present, none of the existing interventions can cure the disease, and patients still have to bear the burden of daily disease management and the risk of complications (13).

The interaction between environmental factors and genetic susceptibility may exacerbate the onset and progression of T1D (14). The growing evidences indicate that intestinal barrier disruption caused by enteropathogen infection, as well as immune responses triggered by dysbiosis, increases the risk of developing T1D (15–17). Given that the gut microbiota serves as an upstream environmental regulator of the immune system, targeting microbial dysbiosis represents a reasonable adjunctive therapeutic strategy. As shown in **Figure 1**, significant progress has been made in the application of probiotics in the treatment of T1D (**Figure 1**). Studies on multiple cohort samples have revealed that pathogenic bacteria and proinflammatory bacteria are significantly enriched in patients with T1D (18). In contrast, probiotics are significantly reduced in T1D patients (19, 20). The beneficial regulation of intestinal microbiota through probiotic administration promotes the proliferation of health-beneficial bacterial strains, which are currently being utilized to advance the development of probiotic therapeutic interventions (21). Research has demonstrated that the use of probiotics, such as *Lactobacillus salivarius AP-32*, *L. johnsonii MH-68*, and *Bifidobacterium animalis CP-9*, can enhance commensal probiotics including *Bifidobacterium* and *Akkermansia muciniphila*, while reducing pathogenic species of *Alistipes*, *Allobaculum*, *Dorea*, *Odoribacter*, *Parabacteroides*, and *Retroviridae* (22).

**Figure 1:**
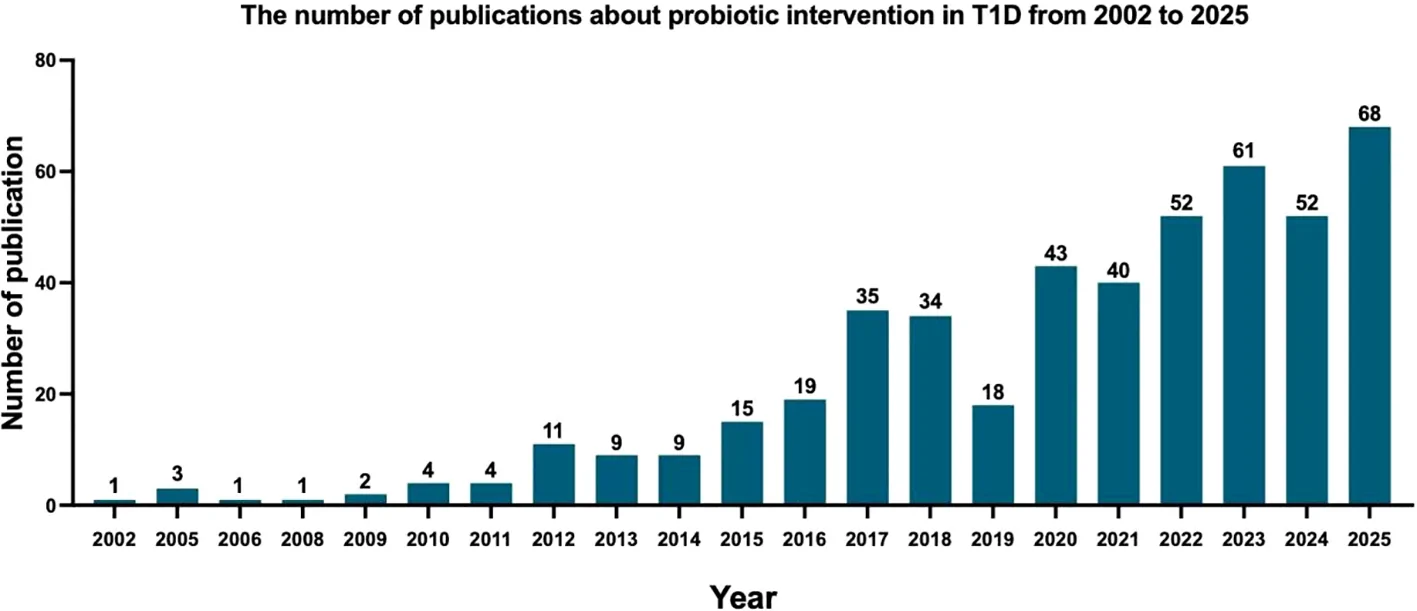
The number of publications about probiotic intervention in T1D from 2002 to 2025 (until December 2025). The data were obtained by searching in Web of Science (https://www.webofscience.com/) on the topic of “probiotics” and “Type 1 diabetes”.

However, to fully exert the immunomodulatory and blood glucose-stabilizing effects of probiotics, they must survive the harsh gastrointestinal environment, including the low pH of the stomach, enzymatic degradation, and the antibacterial activity of bile salts. Therefore, future studies are focused on developing more robust and precise delivery systems or approaches. Meanwhile, they also face heterogeneity in clinical trial results caused by individual differences. This review summarizes the immune-mediated mechanisms of probiotic therapy for T1D, as well as its efficacy, safety, and development prospects in the treatment and adjuvant treatment of T1D. Firstly, it systematically reviews the potential mechanisms of probiotics in the prevention and treatment of T1D, then comparatively analyzes the factors contributing to the discrepancies in current clinical trial results related to probiotic therapy, and also discusses the limitations and advantages of existing probiotic therapeutic strategies. Finally, it looks forward to the future development directions and application prospects of probiotics. Several reviews on similar topics have been published recently, but none have included an analysis of the heterogeneity in clinical trial results, a comprehensive summary of engineered probiotics for T1D treatment, or a specific discussion on the prospects of future research. This review aims to investigate more personalized and targeted probiotic strategies, thereby offering novel perspectives for the prevention and management of T1D (23–27).

## 2 Gut microbiota dysbiosis and T1D

### 2.1 Aberrant gut microbiota in T1D patients

During recent times, numerous studies have identified characteristic intestinal dysbiosis in patients with T1D, which is characterized by the coexistence of reduced beneficial bacteria and expanded opportunistic pathogens (28, 29). Studies on multiple cohort samples have revealed that pathogenic and pro-inflammatory bacteria—including Escherichia coli, Klebsiella pneumoniae, as well as Parabacteroides—are significantly enriched in T1D patients (30–32). In contrast, probiotics such as Bifidobacterium, butyrate-producing bacteria (e.g., *Faecalibacterium prausnitzii*, *Roseburia intestinalis)*, *Prevotella* spp., and *Faecalibacterium* spp. are markedly depleted in these patients (19, 20, 30, 33, 34). Based on the compositional and functional analyses of the gut microbiota, patients with T1D exhibit gut dysbiosis characterized by decreased α-diversity and altered β-diversity clustering (35).

Notably, gut microbial changes in T1D are characterized by shifts at the species and strain levels rather than genus-wide alterations. For instance, while certain strains of Bacteroides may exert protective effects via immunomodulatory metabolites, others may promote inflammation through LPS-enriched outer membrane vesicles (36–38).

### 2.2 Relationship between the gut microbiome and T1D

The autoimmune pathogenesis of T1D involves the synergistic interaction of islet inflammation dominated by cytotoxic effector T cells (CD8^+^ T) cells and macrophages, β-cell abnormalities, and exocrine pancreas dysfunction, which collectively contribute to the destruction of insulin-producing cells and disease progression (39).

The onset of T1D is not a simple combination of isolated events, but a progressive vicious cycle mediated by the gut–immune–pancreas axis. This process involves intestinal microbiota dysbiosis and impaired gut barrier function, which further trigger immune system activation and ultimately lead to pancreatic damage (40) (**Figure 2**).

**Figure 2:**
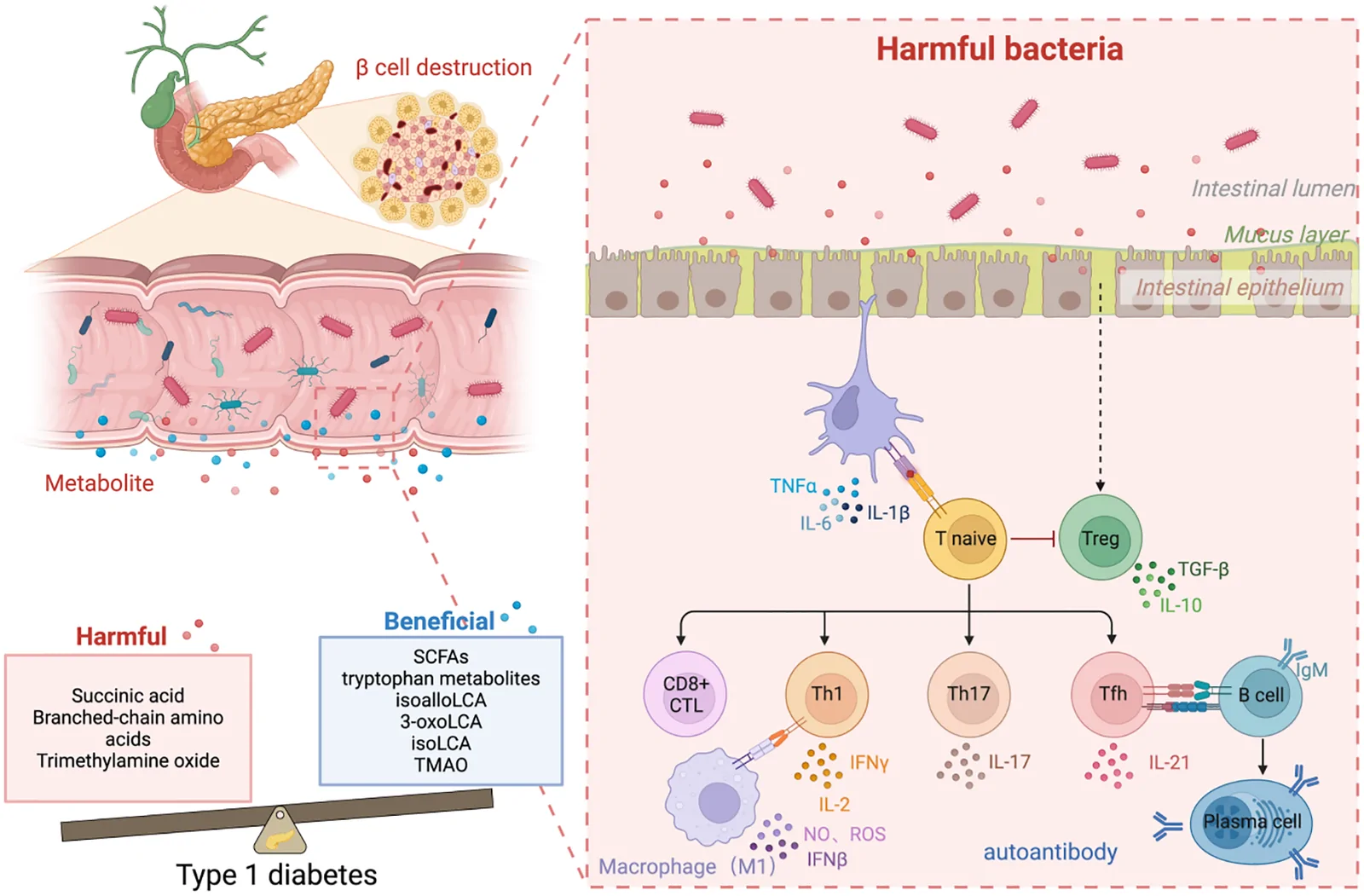
The interactions between the gut, pancreas, and immune system in T1D. Gut dysbiosis causes an imbalance in microbial metabolites. Harmful bacterial metabolites activate immune responses, inducing pro-inflammatory cytokines and autoantibodies that aggravate pancreatic injury.

Intestinal microbiota homeostasis plays a vital role in maintaining host immune homeostasis, and the association between microbiota dysbiosis and immune dysregulation has long been confirmed in immune-mediated disorders such as inflammatory bowel disease (IBD) and allergic asthma (41–43). Intestinal microbiota metabolites act as important mediators in microbiota–immune crosstalk, and their abnormal levels contribute to the disruption of immune homeostasis.

Deficiency of short-chain fatty acids (SCFAs), especially butyrate, represents a crucial link connecting gut microbiota dysbiosis and immune dysregulation. Under physiological conditions, SCFAs such as butyrate not only enhances epithelial barrier integrity to prevent the translocation of harmful substances, but also maintains immune tolerance (44, 45). However, in T1D, the depletion of butyrate-producing bacteria (e.g., *Faecalibacterium prausnitzii*) leads to reduced SCFA levels, which is often accompanied by intestinal barrier disruption and Th17/Treg imbalance. This pathological state is not confined to the gut; instead, it may systemically exacerbate autoimmune attack against the pancreas as well as damage to other organs, such as cerebral ischemic injury (46, 47). Furthermore, dysregulation of aromatic amino acid metabolites is equally crucial. For instance, indole compounds produced by intestinal bacteria regulate mucosal immunity via activating the aryl hydrocarbon receptor (AhR), maintain local immune tolerance, and exert positive effects on promoting Treg differentiation and inhibiting Th17 responses. Reduced levels of these metabolites are closely associated with the development and progression of inflammatory bowel disease and systemic autoimmunity (48, 49). However, under certain genetic backgrounds, specific isolates of *Lactobacillus reuteri* can promote Th17 immune responses via the AhR pathway (50). These disparate results may indicate that the key to determining immunological outcomes lies in strain-level precision rather than genus-level generalizations. Alterations in bile acid metabolism also constitute a key immunomodulatory pathway (51). Bile acids and their secondary metabolites generated by microbial transformation (e.g., isoalloLCA, 3-oxoLCA, and isoLCA) can regulate macrophage polarization and cytokine production via receptors such as FXR and TGR5, thereby influencing immune homeostasis (52). Among these metabolites, isoalloLCA promotes the differentiation of Tregs via the nuclear receptor NR4A1 to enhance immune tolerance, whereas 3-oxoLCA and isoLCA suppress Th17 cell differentiation by inhibiting RORγt (53, 54). In patients with autoimmune diseases such as T1D, the levels of these immunosuppressive bile acid metabolites may decrease, which impairs their “braking effect” on the immune system. In contrast, another class of gut-derived metabolites, such as trimethylamine N-oxide (TMAO), has been proven to activate inflammatory pathways including the NLRP3 inflammasome (55), promote the polarization of immune cells toward a pro-inflammatory phenotype, impair vascular endothelial function, facilitate microcirculatory disorders, and participate in and exacerbate immune-mediated pathological processes such as acute pancreatitis and diabetic nephropathy (56–58).

Antigens derived from the gut, together with the dysregulated immune microenvironment, synergistically initiate the autoimmune response targeting islet antigens. In this process, CD8^+^ T cells are the primary executors that directly destroy pancreatic β-cells. Their differentiation and activation are modulated by the assistance of CD4^+^ T cells and the functional impairment of regulatory T cells (Treg cells) (59). Meanwhile, intrinsic defects of β-cells—including endoplasmic reticulum stress (60–62), mitochondrial dysfunction (63–66), and senescence-associated secretory phenotype (SASP) (67–69)—not only enhance the exposure of self-antigens and activate the innate immune response (70–72), but also trigger a vicious cycle that continuously amplifies immune attack and β-cell damage via the secretion of pro-inflammatory cytokines (73–76). Furthermore, autoantibodies against islet antigens serve as crucial biomarkers of this pathological process and may also participate in driving the immune response in the early stage of the disease (71, 72).

In a word, gut ecological imbalance leads to abnormal activation of the immune system. Activated immune cells then target pancreatic β-cells with inherent susceptibility. The subsequent stress and death of β-cells further provide additional antigens and stimulatory signals to the immune system. This dynamic axis linking the gut, immune system, and pancreatic β-cells drives the irreversible progression of type 1 diabetes from the subclinical to the clinical stage.

## 3 Mechanisms of action of probiotics in T1D

Probiotics protect host health mainly via two pathways: preserving intestinal barrier integrity and regulating the host immune system. The barrier acts as the first line of defense against pathogens, while immune modulation maintains homeostasis by balancing immune and inflammatory responses. In the following sections, the specific mechanisms of probiotics will be elaborated in detail from the two aspects of intestinal barrier protection and immune regulation.

### 3.1 Intestinal barrier protection

Disruption of intestinal barrier integrity leads to the activation of islet-specific T cells in the intestinal mucosa, which in turn triggers autoimmune diabetes (74). Impaired intestinal mucosal barrier function facilitates the translocation of bacteria and their metabolites across the epithelial barrier into the circulation. Increased intestinal permeability often precedes the onset of pancreatic islet inflammation (75, 76), suggesting that it may serve as an early trigger of the disease.

Probiotics can directly repair the barrier structure. For instance, in NOD mouse models, the combined use of prebiotics (e.g., inulin) and probiotics along with omega-3 polyunsaturated fatty acids (PUFAs) restores intestinal barrier function. It also indirectly modulates intestinal pH by regulating gut microbiota homeostasis, thereby reducing pancreatic islet inflammation and delaying T1D onset (77). Second, their metabolites, SCFAs, actively reinforce the barrier. SCFAs produced by probiotics can regulate the NF-κB pathway via activating G protein-coupled receptor 43 (GPR43) and inhibit histone deacetylases (HDACs), thereby maintaining intestinal barrier integrity and reducing bacterial translocation (78).They promote the proliferation of intestinal epithelial cells and strengthen tight junctions, thus alleviating T1D-associated intestinal barrier damage (79). In addition, foods rich in fermentable fibers (e.g., pectin) are metabolized by probiotics to generate abundant SCFAs, which improve intestinal permeability and immune homeostasis (80).

Specific probiotic strains, such as *Faecalibacterium prausnitzii* and *Akkermansia muciniphila*, exert significant immunomodulatory effects in T1D and further regulate intestinal pH via SCFA production. *F. prausnitzii* enhances intestinal barrier integrity by generating SCFAs and secreting microbial anti-inflammatory molecules (MAMs), whereas *A. muciniphila* modulates mucosal immunity by activating Toll-like receptor 2 (TLR2) and strengthening tight junctions (81). More interestingly, probiotics can also optimize the chemical properties of the barrier. Studies have shown that reduced phosphatidylcholine (PC) levels are observed in the intestinal mucus layer of T1D mouse models—particularly a marked decrease in phosphatidylcholine in the colonic mucus layer. This may impair the hydrophobicity and barrier function of the mucus layer. Probiotics, however, may indirectly modulate mucus components by regulating microbiota balance, optimize the chemical properties of the mucus, and consolidate this line of defense (82).

However, barrier repair at the level of specific strains only represents a form of “single-point reinforcement”. Improvement in intestinal barrier function is also associated with the diversity of the gut microbiota and changes in the abundance of specific microbial taxa. For example, in the gut microbiota of T1D patients, the abundances of *Blautia* and *Akkermansia* are abnormal, and these alterations are correlated with increased intestinal permeability and cognitive decline (83). Impaired intestinal barrier function is linked to decreased expression of mucins such as MUC2 and MUC12 as well as antimicrobial peptides such as HD5 and HD6 in the mucus layer, along with reduced abundances of SCFA-producing bacteria including *Bifidobacterium* and *Roseburia* (84). By regulating the gut microbiota and enhancing the expression of tight junction proteins ZO-1 and claudin-1, probiotics can significantly reduce intestinal permeability, thereby decreasing bacterial translocation and systemic inflammation (85). Changes in the abundance of specific microbiota synergistically amplify intestinal barrier defense signals through collective metabolic output rather than the action of individual strains.

In summary, probiotics can lower intestinal pH and enhance the expression of tight junction proteins, thereby improving mucosal integrity, preventing bacterial products (e.g., lipopolysaccharide, LPS) from entering the systemic circulation, and suppressing the consequent inflammatory responses (**Figure 3**).

**Figure 3:**
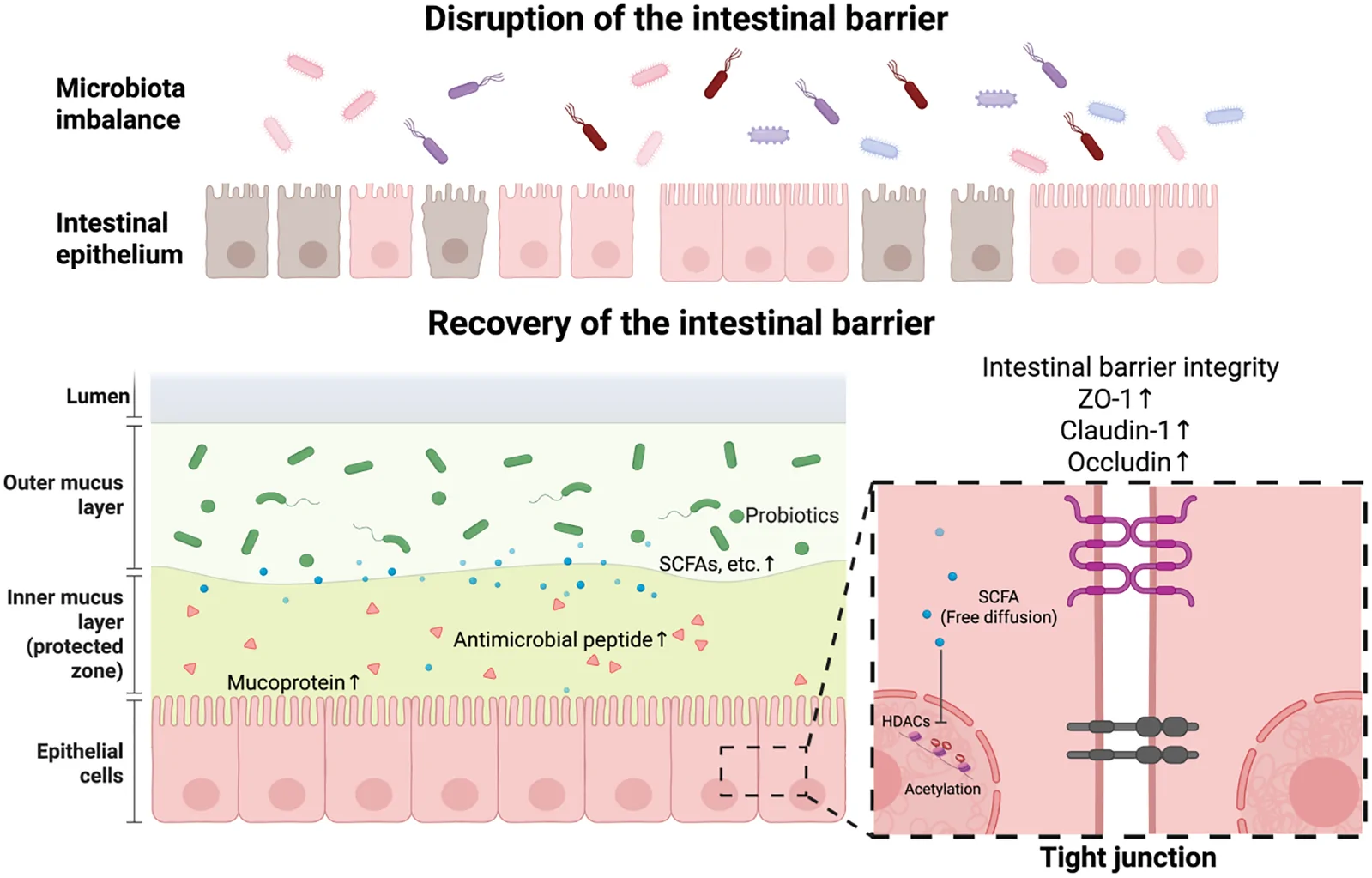
The mechanism of probiotics in repairing intestinal barrier in T1D. Probiotics produce beneficial metabolites including SCFAs,etc., upregulate mucins and antimicrobial peptides, enhance tight junction proteins such as ZO-1 and claudin-1, thereby reducing intestinal permeability and repairing intestinal barrier damage in T1D.

### 3.2 Direct immune regulation

Beyond reinforcing the structural integrity of the intestinal epithelium, recent animal studies indicate that probiotics exert direct immunomodulatory effects by converging on key processes, including T cell differentiation, microbial metabolite utilization, and innate immune regulation (**Figure 4**).

**Figure 4:**
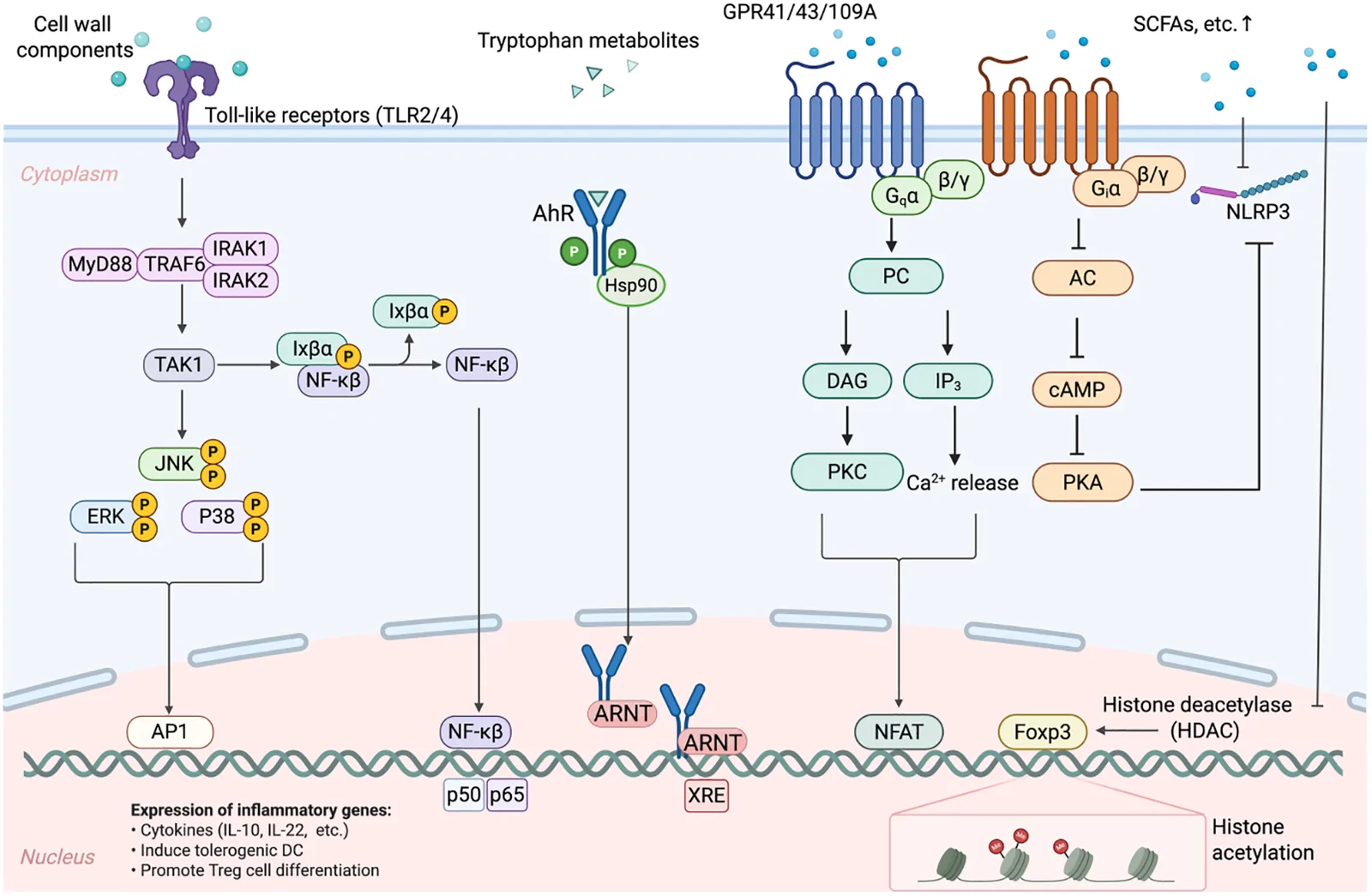
Immune mechanism of probiotics in T1D. Probiotics regulate immune cell differentiation via multiple signaling pathways, including SCFAs-GPCR/HDAC, TLR-MyD88, and AhR-XRE. This ultimately leads to Treg expansion, suppression of Th1/Th17 cells and inflammasomes, decreased pro-inflammatory cytokines, and increased anti-inflammatory cytokines.

The core protective mechanism of various probiotics lies in promoting the differentiation of Tregs that mediate immune tolerance, while simultaneously suppressing pro-inflammatory Th17 or Th1 cells. For example, the probiotic *VSL#3* modulates the gut microbiota and shapes a tolerogenic intestinal microenvironment enriched with indoleamine 2,3-dioxygenase (86). Some bacterial strains can upregulate IDO expression to generate immunosuppressive metabolites, thereby further promoting immune tolerance (87). This not only significantly enhances Treg differentiation and upregulates the anti-inflammatory cytokines IL-10 and TGF-β1, but also inhibits the activity of pro-inflammatory Th17 cells and their associated cytokines IL-17 and TNF-α, thereby ameliorating pancreatic islet autoimmunity and delaying the progression of T1D. Similarly, *Lactobacillus johnsonii N6.2* (*LjN6.2*), a strain isolated from diabetes-resistant rats, significantly reduces the incidence of T1D in diabetes-susceptible rats by inducing the skewed differentiation of Th17 cells (88). Notably, the increase in Th17 cells occurs primarily in mesenteric lymph nodes, indicating that the gut microbiota can modulate systemic immune responses via local intestinal immune regulation. Furthermore, *LjN6.2* enhances Th17 differentiation through antigen-presenting cells (APCs) and induces dendritic cells (DCs) to adopt an immature phenotype with migratory regulatory properties, thereby exerting a protective effect (88, 89). Multiple animal studies have demonstrated that probiotic supplementation markedly increases the proportion of FoxP3^+^ Tregs in pancreatic and intestinal lymph nodes, while inhibiting the expansion of Th1/Th17 cells (90). This leads to the upregulation of IL-10 and TGF-β, along with the downregulation of IL-17 and IFN-γ, reflecting an overall shift in the cytokine profile toward an anti-inflammatory phenotype (91–93).

Microbial metabolites act as key messengers mediating immunomodulation. A gluten-free diet increases the abundance of butyrate-producing probiotics such as *Roseburia* and *Clostridium butyricum*, thereby elevating SCFA levels, significantly reducing pancreatic islet inflammation and diabetes incidence in NOD mice (94), and synergistically modulates the antioxidant defense and endoplasmic reticulum (ER) stress responses of pancreatic β-cells in combination with *Lactiplantibacillus plantarum 299v* (*Lp299v*) (95). Additionally, human milk oligosaccharides (HMOs), as microbiome modulators, delay the progression of T1D by increasing the number of probiotics, upregulating SCFA levels, and inducing tolerogenic dendritic cells (tDCs) (96). The probiotic *AKK* and butyrate exert a synergistic therapeutic effect on glycemic reduction, metabolic regulation, and immunomodulation in T1D models by cooperatively regulating gut microbiota composition, enhancing SCFA production (e.g., butyrate and propionate), and alleviating systemic inflammation as well as pancreatic β-cell damage (97). Furthermore, the combined probiotics *Clostridium butyricum CGMCC0313.1* and *AKK* have been shown to promote Treg migration to the pancreas via SCFAs they produce, thus restoring local immune homeostasis (90). However, the gut microbiota of T1D patients is often characterized by reduced diversity and depletion of SCFA-producing bacteria such as *Prevotella* and *Butyricimonas*, which correlates with increased intestinal permeability and systemic inflammation (98). Notably, antibiotic use disrupts microbiota homeostasis, reduces SCFA production, accelerates T1D onset in NOD mice, and is accompanied by Th1/Th17 imbalance (99). Although oral butyrate supplementation exerts a protective effect in animal models, it fails to improve immune parameters in patients with long-standing T1D (100). This suggests that microbiota-immune axis-based interventions have a strict “time window”, and early intervention may be crucial. Beyond SCFAs, α-galactosylceramide (α-GalCer), a sphingolipid produced by Bacteroides fragilis, exerts a protective effect by activating natural killer T (NKT) cells and anti-inflammatory M2 macrophages, significantly reducing T1D incidence in NOD mice. This protective effect depends on α-GalCer produced by viable bacteria rather than dead bacteria, highlighting the critical role of specific metabolites in immunomodulation (101).

Probiotic-mediated modulation of DCs and inhibition of innate immune inflammation also serve as another critical immunomodulatory mechanism. Probiotics can guide immune tolerance at the source by regulating the phenotype and function of APCs, characterized by the downregulation of MHC-II and CD80/CD40 as well as the upregulation of ICAM-1 (36). *Lactobacillus rhamnosus GG (LGG)* can induce intestinal CD103^+^ dendritic cells to differentiate toward a tolerogenic phenotype, downregulate their expression of co-stimulatory molecules and pro-inflammatory cytokines (IL-12, TNF-α), and promote IL-10 production. This shifts antigen presentation toward the induction of Tregs rather than pathogenic effector T cells (102). In addition, probiotics can suppress the activation of the downstream MyD88-dependent NF-κB signaling pathway by regulating the expression of intestinal Toll-like receptors (TLR2/4), thereby fundamentally reducing the production of pro-inflammatory cytokines such as IL-1β and TNF-α (103). Probiotic-induced gut-derived IL-10^+^ lymphocytes can circulate to pancreatic lymph nodes, suppress the presentation of autoantigens, and protect residual β-cells (104). Meanwhile, probiotic-stimulated secretion of glucagon-like peptide-1 (GLP-1) also indirectly enhances islet function and inhibits inflammation, which may be associated with the upregulation of the PI3K/AKT pathway (105).

## 4 Clinical studies of probiotics in the treatment of T1D

Although animal models and ex vivo studies have elucidated sophisticated molecular pathways, the direct translation of these pathways into reproducible clinical benefits in humans remains tenuous. The efficacy of probiotics in delaying or preventing T1D has exhibited marked heterogeneity across multiple human clinical trials, underscoring that animal findings should not be equated with clinical benefit. Emerging evidence indicates that specific probiotics exert potential benefits in preserving pancreatic β-cell function, slowing the decline of C-peptide levels, and modulating immune-inflammatory markers among high-risk children with positive islet autoantibodies or newly diagnosed patients. Nevertheless, a considerable number of clinical trials have failed to replicate such significant protective effects. An in-depth analysis of these studies reveals that the discrepancies in outcomes primarily stem from differences in intervention timing, strain specificity, and core outcome measures. A summary of clinical studies of probiotics in the treatment of T1D is listed in **Table 1**.

**Table 1:** Clinical trial study of probiotics in the treatment of T1D.

Probiotics	Dosage	Target population & timing	Sample size	Follow-up duration	Outcome measures	Ref.
*Visbiome®*	Once daily; 11.25 billion (11.25 × 10^9) CFU/capsule (lyophilized)	Children aged 2–12 with newly diagnosed T1D (within 6 months of onset)	90	3 months	HbA1c, C-peptide, IR, Glycemic variability, Remission rate	(106)
*Vivomixx®*	Once daily; 22.5 billion (22.5 × 10^9) CFU/sachet (lyophilized powder)	Children aged 2–12 with newly diagnosed T1D (within 6 months of onset)	50	6 months	HbA1c, C-peptide, IR, IMMs (Treg percentage, IL-10 ), autoantibodies (IAA, IA-2, GAD65), FBG, TBG,	(107)
*Bifidobacterium longum subspecies infantis EVC001*	Once daily;≥8×10^9 CFU/sachet (actual batch: 13.8–15.8×10^9 CFU)	Genetically high-risk infants (administered from 7 days to 6 weeks postpartum)	1144	12 months	Time to appearance of β-cell autoantibodies (IAA, GAD65, IA-2, ZnT8)	(108)
*Bifidobacteria, Lactobacillus*, and *Streptococcus* strains	Once daily; total dosage not specified	Siblings of children with T1D, aged 6.2–17.7 years	25	6 weeks	Systemic inflammation index; GMD, SBA, Firmicutes/Bacteroidetes ratio; short-chain fatty acid levels; immune cell ratio [CD4+ memory (CD45RO+) vs. naive (CD45RA+) T cells]	(109)
*Lactobacillus GG (ATCC 53103), Lactobacillus rhamnosus LC705 (DSM 7061), Bifidobacterium breve Bb99 (DSM 13692), Propionibacterium freudenreichii ssp. shermanii JS (DSM 7076)*	ATCC 53103: 5 × 10^9^ CFU/day; L. DSM 7061: 5 × 10^9^ CFU/day;DSM 13692: 2 × 10^8^ CFU/day; DSM 7076: 2 × 10^9^ CFU/day	Infants with high risk of allergy	1223	From birth to 13 years	Incidence rate; autoantibodies (IAA, GAD65, IA-2, ZnT8); upper respiratory tract infection (URTI) frequency; antibiotic use frequency; duration of exclusive and total breastfeeding	(110)
*Lactobacillus rhamnosus GG, Lactobacillus johnsonii N6.2*	10 drops daily (divided into two doses, 5 drops each), 1 × 10^9^ CFU/drop	Children and adolescents with T1D aged 3–18 years	79	6 months	Influenza vaccine immunogenicity (hemagglutination inhibition antibody titer, seroconversion rate, seroprotection rate); IMMs (IFN-γ, IL-17A, IL-6, TNF-α, etc.); immune cells (flow cytometry analysis of CD4^+^, CD25^+^, Foxp3^+^ T cells)	(111)
*Lactobacillus johnsonii N6.2*	Once daily;1 × 10^9^ CFU/day	Healthy adults (without T1D risk factors)	42	3 months	Immune cells (flow cytometry analysis of monocytes, dendritic cells, etc.); tryptophan metabolism (serum tryptophan, kynurenine levels, and T: K ratio)	(112)
*Lactobacillus rhamnosus GG, Bifidobacterium breve Bb12*	L. rhamnosus GG: 0.5 × 10^9^ CFU/day; B. breve Bb12: 0.5 × 10^9^ CFU/day	Genetically high-risk infants (HLA-DQB10302 and/or HLA-DQB10201 genotype)	200	6 months	Incidence of β-cell autoantibodies (GADA, IA-2A, IAA); reduction in enterovirus infections	(113)
*Lactobacillus reuteri DSM 34531, Lactobacillus rhamnosus DSM 26357, Lactobacillus lactis DSM 32299*	Initial total dose: 7.5 × 10^9^ CFU/day (divided into two doses);9.3 × 10^8^ CFU/day (due to storage loss)	Patients with T1D and albuminuria	35	30 weeks	UACR; renal function [glomerular filtration rate (^99^mTc-DTPA clearance)]; 24-hour systolic blood pressure; endothelial function [glycocalyx thickness (GlycoCheck value)]; IMMs [TNF-α, IL-6, IL-8, IL-1β]; intestinal barrier function [serum LPS activity, fatty acid-binding protein (FABP2)]	(114)
*Lactobacillus rhamnosus GG, Bifidobacterium breve Bb12*	Once daily; 1 × 10^9^ CFU/day	Newly diagnosed T1D children	96	12 months	C-peptide; HbA1c; IR	(115)
*Lactobacillus salivarius subsp. salivarius AP-32, Lactobacillus johnsonii MH-68, Bifidobacterium animalis subsp. lactis CP-9*	Once daily; total dosage not specified	Patients with T1D aged 6–18 years	96	9 months	FBG; HbA1c; IMMs (IL-8, IL-17, MIP-1β, RANTES, TNF-α, TGF-β1); GMD; SBA	(116)

FBG, fasting blood glucose; TBG, total blood glucose; UACR, urinary albumin-to-creatinine ratio; IR, insulin requirement (total, basal, and bolus doses); IMMs, immunomodulatory markers; GMD, gut microbiota diversity; SBA, specific bacteria abundance.

### 4.1 Intervention timing may be a decisive factor

The potential efficacy of probiotics in T1D may depend on the stage at which the intervention is initiated; however, preventive intervention before disease onset and therapeutic intervention after T1D diagnosis should be considered as distinct clinical scenarios.

In patients with newly diagnosed T1D, probiotic intervention resulted in a significant reduction in glycated hemoglobin (HbA1c) levels and insulin requirements, with some achieving disease remission. However, no improvement in C-peptide levels was observed, suggesting that the protective effect may be mediated by the preservation of residual β-cell function (indicated by C-peptide levels) (106, 107). These findings provide stronger support for investigating probiotics during the early post-diagnosis period than for assuming that probiotics are equally effective throughout the entire course of T1D. However, these studies should be interpreted as evidence for a potential early therapeutic window, rather than proof that probiotic treatment must be initiated immediately after diagnosis or that earlier treatment is universally more effective. The rationale for this therapeutic window is biologically plausible because early after diagnosis, residual β-cell function may still be detectable, whereas progressive autoimmune-mediated β-cell destruction may reduce the opportunity for interventions aimed at preserving endogenous β-cell function.

Importantly, the evidence for primary prevention is substantially less conclusive. In individuals at genetic or immunological risk before clinical onset, the potential value of probiotics may instead depend on whether intervention occurs before substantial autoimmune activation and β-cell destruction (108, 109). In infant intervention trials, a multi-strain probiotic formulation containing *LGG* failed to significantly reduce the positivity rate of islet autoantibodies or the incidence of T1D (110). Preventive trials in genetically predisposed infants have not consistently demonstrated reductions in islet autoantibody seroconversion or T1D incidence, indicating that early administration alone does not guarantee clinical benefit. A meta-analysis revealed that probiotics conversely exacerbated glycemic control in pediatric and adolescent T1D patients, leading to elevated HbA1c and fasting blood glucose levels (117). Further emphasizing that age, pubertal status, disease stage, and metabolic background may modify probiotic responses.

During early stages of disease development, including genetically susceptible individuals or those with islet autoantibody positivity, modulation of intestinal immunity may theoretically influence immune tolerance before extensive β-cell destruction occurs. In contrast, after the establishment of autoimmune responses and substantial β-cell loss, probiotic supplementation alone may be insufficient to reverse disease progression. Future trials should directly compare preventive and post-diagnosis intervention windows and incorporate clinically relevant endpoints, including islet autoantibody conversion, C-peptide preservation, HbA1c, insulin requirements, and immune or microbiome-related biomarkers, to determine whether a reproducible temporal window for probiotic efficacy exists.

### 4.2 Strain specificity and dosage do not confer equivalent effects

Fundamental differences exist in the bacterial species and even strain combinations used across different studies. The clinical effects of probiotics in T1D appear to be strongly strain- and formulation-dependent, and therefore the term “probiotics” should not be regarded as a biologically homogeneous therapeutic category.

For example, administration of Lactobacillus rhamnosus GG (LGG) together with Bifidobacterium lactis Bb12 at 10^9 CFU/day for 6 months did not significantly improve stimulated C-peptide or other major clinical outcomes in children with newly diagnosed T1D, despite both strains having previously demonstrated probiotic activity in other clinical settings (111). By contrast, a high-potency multi-strain formulation containing approximately 112.5 billion viable bacteria per capsule was associated with lower HbA1c and reduced total and bolus insulin requirements in children with new-onset T1D after 3 months of treatment (106). These findings should not be interpreted as evidence that a higher bacterial dose is intrinsically superior, because the intervention differed from the LGG/Bb12 trial not only in dose but also in strain composition, formulation, patient age, disease stage, and treatment duration.

At the strain level, Lactobacillus johnsonii N6.2 provides another example of potentially distinct biological activity, as this isolate was reported to modulate tryptophan metabolism and immune-cell profiles in a randomized trial, suggesting that metabolic and immunological functions may vary substantially even among closely related probiotic strains (112). The apparent superiority of some strains or combinations may therefore reflect differences in their ability to interact with the host intestinal environment, modulate epithelial barrier function, alter microbial metabolites such as short-chain fatty acids or tryptophan-derived metabolites, and influence regulatory and effector immune-cell responses; however, these mechanisms have not yet been established as definitive predictors of clinical efficacy in T1D. Future research should focus on specific strains with well-defined immunomodulatory mechanisms.

Dosage, whether high or low, may also exert a certain impact on the outcomes. High-dose, multi-strain probiotic interventions such as those with *Visbiome^®^* (containing 112.5 billion CFU per day) and Vivomixx^®^ have been shown to yield metabolic benefits (106, 107), whereas low-dose probiotic interventions may be insufficient to produce significant effects (113). Relevant studies have also found that a 12-week synbiotic (probiotics + prebiotics) intervention failed to significantly improve the urinary albumin-to-creatinine ratio (UACR) and inflammatory markers in T1D patients with proteinuria, which may be attributed to the specific strain combination used or insufficient dosage (114). However, the favorable findings observed with high-strength formulations cannot establish that doses exceeding 10^11 CFU/day are required for therapeutic efficacy, because these studies did not directly compare multiple doses of the same formulation (106, 107).

Taken together, the current evidence supports a strain- and formulation-specific rather than a simple dose-dependent model of probiotic efficacy in T1D. The optimal probiotic dose for T1D therefore remains unknown, and no evidence-based universal CFU threshold can currently be recommended across strains, age groups, or disease stages.

### 4.3 Differences in evaluation metrics and mechanisms

There are significant discrepancies in the endpoint metrics focused on across different studies. For example, in some studies where the incidence of β-cell autoantibodies (GADA, IA-2A, IAA) was set as the primary endpoint, probiotic interventions such as *LGG* were found to exert no significant effect on the autoantibody positivity rate in high-risk infants (113). In contrast, other studies that focused on immunomodulatory indices (e.g., the proportion of induced regulatory T cells [iTregs] and IL-10 levels) demonstrated that high-dose probiotics (*Vivomixx^®^*) significantly increased iTreg counts (p=0.034) and reduced HbA1c levels (p=0.017) (107). Additionally, some studies with glycemic control parameters (HbA1c) and insulin dosage as core metrics reported that high-dose multi-strain probiotics (*Visbiome^®^*) could decrease insulin requirements (p=0.037) (106). Intervention studies involving children with newly diagnosed T1D showed that short-term supplementation (e.g., for 6 months) with *LGG* and *Bifidobacterium lactis Bb12* failed to improve β-cell function (i.e., no changes in C-peptide levels). However, enhanced thyroid autoimmunity was observed, and fluctuations in HbA1c levels and insulin requirements occurred after the discontinuation of intervention (115, 116).

These discrepancies suggest that the effects of probiotics may vary depending on the evaluation metrics used, and such heterogeneity may reflect that probiotics function via distinct pathways. For newly diagnosed T1D, a practical core set should include HbA1c, stimulated C-peptide, daily insulin requirements, and clinically relevant measures of glycemic variability, while immune and microbiome-related biomarkers should be prespecified as secondary or exploratory outcomes (106, 107). For prevention trials in genetically or immunologically high-risk individuals, time to islet autoantibody seroconversion and progression to clinical T1D should be prioritized, with immune and microbiome biomarkers serving as supportive mechanistic endpoints (108, 110). Immune and microbiome-related biomarkers, such as IL-10, inflammatory cytokines, and changes in gut microbial composition, may provide mechanistic information, but they should be considered complementary rather than substitutes for clinically relevant metabolic or disease-related outcomes (111, 114). Overall, the current evidence suggests that the major challenge is not simply the lack of outcome measures, but the absence of a consistent hierarchy linking patient-centered clinical endpoints with β-cell, immune, and microbiome-related biomarkers.

### 4.4 Methodological confounders in current clinical evidence

In addition to the above, three intrinsic methodological confounders also warrant rigorous re-examination. First, small sample sizes—typically 20 to 50 participants per group in most exploratory studies—limit statistical power, particularly for detecting subtle but clinically meaningful changes in C-peptide or islet autoantibody conversion rates (107, 109). Smaller samples inherently constrain subgroup analyses, have low sensitivity for continuous variables such as C-peptide, and increase the risk of type II errors (false negatives). This may explain why multiple trials have reported significant immunomodulatory changes (e.g., iTreg proportions, IL-10 levels) but failed to simultaneously show improvements in glycemic or β-cell functional endpoints. Therefore, future phase IIb/III studies must perform rigorous sample-size calculations based on the minimal clinically important difference for C-peptide (≥0.1 nmol/L).

Second, follow-up durations vary widely—from 6 weeks to 12 months (109, 115). The immunomodulatory effects of probiotics may operate across different temporal phases. Short-term interventions may primarily affect intestinal barrier integrity, inflammatory cytokine levels, and immune cell proportions, whereas protection of β-cell function (e.g., maintenance of C-peptide levels) and delay of disease progression likely require longer follow-up. Consequently, discrepancies in follow-up periods across clinical trials may lead to divergent conclusions regarding probiotic efficacy.

Third, given that genotypes such as HLA-DQ2 and HLA-DQ8 significantly influence antigen presentation, immune activation thresholds, and T1D susceptibility, genetic background may affect individual responses to microbiota-directed interventions. For individuals carrying high-risk genotypes, such as HLA-DRB1*03:01/*04:01/DQB1*02:01/03:02, the gut microbiome already shows reduced α-diversity and impaired butyrate production from infancy. Within this subgroup, the effects of probiotics appear to be largely outweighed by strong genetic susceptibility, which accounts for the null findings in primary prevention trials. In contrast, for those with intermediate- or low-risk genotypes or protective haplotypes (e.g., HLA-DQB1*06:02), the immune system exhibits higher phenotypic plasticity (118, 119). We therefore propose that probiotic intervention is more likely to produce discernible immunometabolic benefits, especially when initiated at the periontogenic stage of metabolic dysregulation, given that the genetic “threshold” for autoimmune development is set higher in these individuals. However, most current clinical trials have not implemented systematic HLA stratification, which may obscure potential responders within overall analyses and further increase inconsistency among study results.

### 4.5 Limitations of current evidence

Although mechanistic and preclinical studies suggest that probiotics may modulate gut microbiota, immune responses, and β-cell-related pathways in T1D, these findings cannot be directly interpreted as evidence of clinical efficacy. Human clinical studies have produced inconsistent results, with some trials reporting improvements in HbA1c, insulin requirements, C-peptide, or immune markers, whereas other studies have failed to demonstrate significant benefits in β-cell function or diabetes-related outcomes (106, 107, 110, 111). This heterogeneity, together with differences in probiotic strains, intervention timing, dosage, treatment duration, and outcome measures, limits the comparability of existing studies and prevents firm conclusions regarding clinical efficacy. Therefore, probiotics should currently be regarded as a promising but investigational adjunctive approach in T1D rather than an established disease-modifying therapy.

The heterogeneity observed in current clinical trials does not negate the value of probiotics; instead, it reveals the complexity of immune interventions for T1D. Future adequately powered, standardized, and longer-term randomized controlled trials are needed to determine whether the biological effects observed in mechanistic and preclinical studies can translate into reproducible and clinically meaningful benefits in patients with T1D.

## 5 The application strategies of probiotics in T1D

Over the past few years, the application of probiotics in the treatment of T1D has shown broad prospects, with significant advancements achieved particularly in studies on engineering modification, delivery systems, and personalized medicine. (**Figure 5**) A summary of these mechanisms for engineered probiotics is listed in **Table 2**.

**Figure 5:**
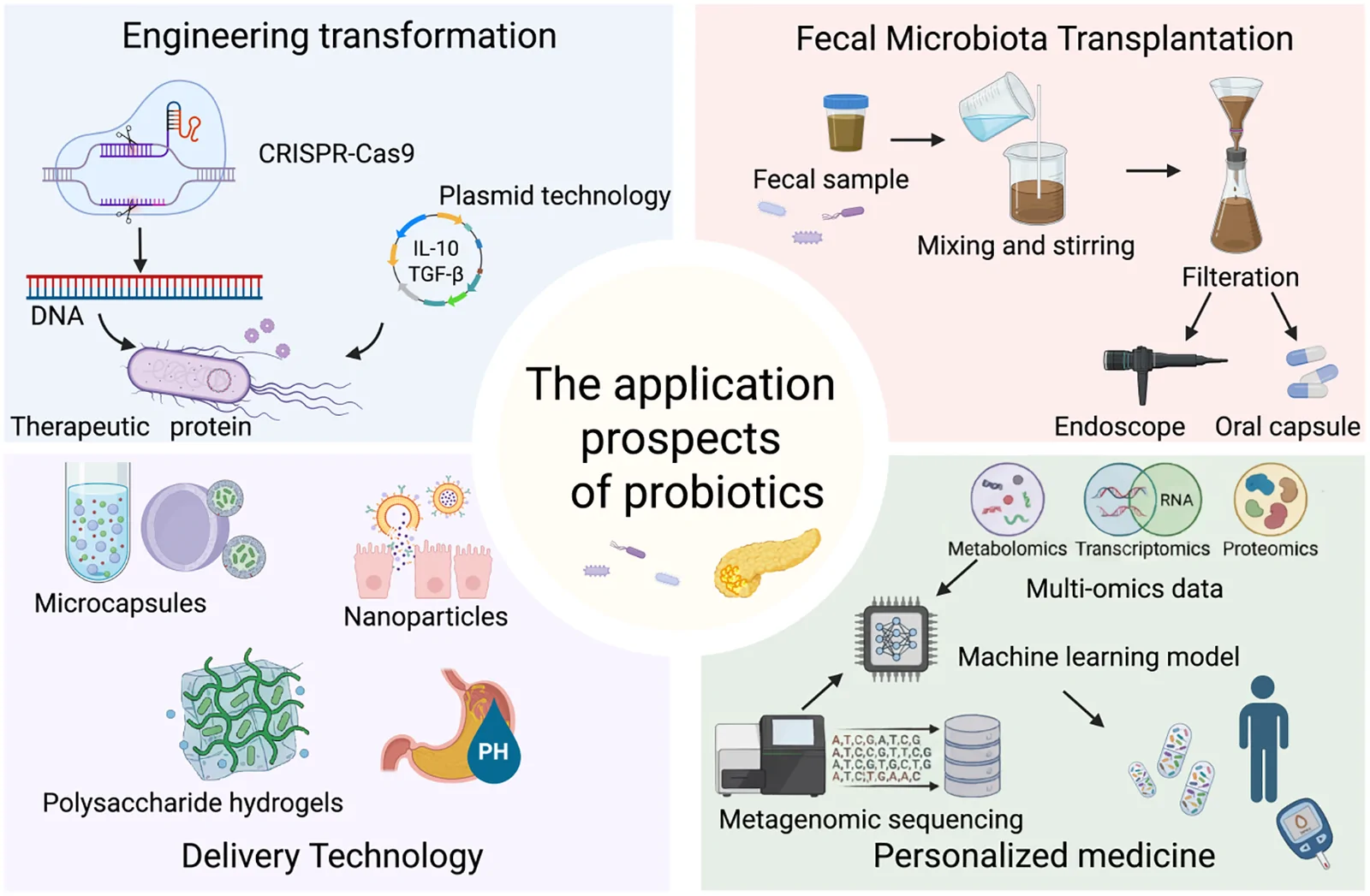
The application strategies of probiotics in T1D.

**Table 2:** Engineered probiotics in treatment of T1D.

Items	Effects on T1D	Mechanisms	Refs.
*L. lactis* expressing therapeutic proteins and autoantigens	Secrete autoantigens and reduce autoimmune attacks	*L. lactis* (secreting proinsulin + IL-10)+Anti-CD3 antibody→CD4^+^CD25^+^FOXP3^+^ Tregs cell↑	(120)
*L. lactis* expressing heat shock protein 65 (HSP65) and tandemly repeated IA2P2 peptide	Improve glucose tolerance and reduce insulitis	*L. lactis* (expressing HSP65-6IA2P2)→IL-10 ↑, IFN-γ ↓, Th1 ↓, Th2 ↑, Th17 ↓, Tregs ↑	(121, 122)
*L. lactis* secreting trimmed GAD65 (370–575) and IA-2 (635–979) fragments, co-expressed with IL-10	Restore tolerance to β-cell autoantigens	*L. lactis* (secreting GAD65_370_–_575_ or IA-2_635-979_ +IL-10)→IFN-γ ↓, FOXP3^+^ Tregs cell↑	(123)
*L. lactis* expressing staphylococcal nuclease (SNase)	Reduce blood glucose, delay diabetes onset, Protect β-cells and reduce inflammation	*L. lactis*(expressing SNase)→neutrophil extracellular traps (NETs)↓, neutrophil elastase(NE)↓, proteinase 3(PR3)↓, IL-4↑, TNF-α↓, zonuli↓, LPS↓	(124, 125)
*qaLactococcus lactis* expressing CRAMP	Correct gut dysbiosis, protect β-cells, improve Metabolic Parameters	*L. lactis*(expressing CRAMP)→tight junction proteins (e.g., ZO-1, occludin)↑, IL-1β↓, IL-18↓, CD86^+^cDC↓s, IFN-γ^+^T cells↓, Foxp3^+^Tregs↑	(126, 127)
*P. pentosaceus* delivering single-chain insulin analog (insulin-CBT1)	Promote β-cell proliferation and enhance glucose uptake	*P. Pentosaceus*(expressing insulin-CBT1)→INSR↑, AKT/ERK/mTOR Pathways↑, β-cell proliferation↑	(128)
*E. coli Nissle 1917* expressing GLP-1	Reduce blood glucose, protect β-cells, and reduce inflammation	*E. coli Nissle 1917* (expressing GLP-1)→TLR-4/NF-κB pathway↓, Bax/Bcl-2 ratio↓, modulates gut microbiota (Bacteroides↓, Helicobacter↓)	(129)
*AG019* (*L. lactis* expressing human proinsulin and IL-10)	improve Metabolic Parameters and reduce insulin need	*L. lactis* (expressing human proinsulin and IL-10)→CD8+ T cells↓, Tregs↑ IL-10↑	(130)

### 5.1 Precision engineering: prospects for the development of engineered probiotics

Lactic acid bacteria (LAB) are widely used for delivering therapeutic molecules due to their safety and intestinal colonization capacity. The application of engineered lactococci (e.g., *Lactococcus lactis*) in T1D treatment—such as strains secreting proinsulin or anti-inflammatory cytokines (e.g., IL-10)—can induce immune tolerance and delay disease progression in mouse models (120). Alternatively, recombinant expression of antigenic proteins such as HSP65-6IA2P2, GAD65_370-575_, and IA-2_635-979_ enables sustained antigen delivery at the intestinal mucosa, maintains immune epitope exposure in NOD mice, and alleviates pancreatic autoimmune damage (121–123). Engineered lactococci can also express staphylococcal nuclease (SNase) targeting neutrophil extracellular traps (NETs), thereby mitigating early autoimmune damage to pancreatic β-cells and regulating blood glucose levels as well as inflammatory responses (124, 125). *Lactococcus lactis* engineered to express CRAMP can reshape the gut microbiome, suppress intestinal inflammation and the activation and migration of IFN-γ+ T cells to the pancreas, thereby providing a novel therapeutic strategy for the prevention or delay of T1D (126, 127).

Recent studies have also begun to explore other bacterial strains, constructing engineered Pediococcus pentosaceus capable of expressing and secreting a single-chain insulin analog (insulin-CBT1). This bacterial delivery system not only preserves the α-helical structure of insulin-CBT1 to enhance its *in vitro* bioactivity but also enables oral delivery, providing a new approach for developing novel probiotic-based therapeutic strategies for diabetes (128). Currently, candidate products such as *Escherichia coli Nissle 1917* (*EcN*) have entered early-phase preclinical trials. Loading insulin, GLP-1, or anti-inflammatory peptides onto the surface or into the cytoplasm of EcN allows for glucose-dependent insulin release after oral administration, significantly reducing blood glucose levels and improving islet function in NOD mice (131). Genetic editing to express immunomodulatory molecules such as GAD65, Insulin-B chain, or IL-35, combined with co-expression of IL-10 or TGF-β, can form a local immune microenvironment in the intestine, promoting Treg expansion and inhibiting autoantibody production (129). This suggests a feasible strategy for transitioning from microecological regulation to live bacterial gene therapy.

In addition, a recent study integrated expression cassettes of human proinsulin and the anti-inflammatory cytokine IL-10 into the chromosome of L. lactis using CRISPR-Cas9 or traditional plasmid technology. Sustained antigen secretion in intestinal epithelial cells after oral administration induces antigen-specific Tregs and achieves immune tolerance. This strain achieved a 66% cure rate in NOD mice and has entered the phase Ib/IIa clinical trial stage (designated AG019), demonstrating favorable safety and preliminary efficacy (130). With the sole exception of AG019, all currently reported engineered probiotic studies remain at the preclinical stage in animal models.

Nevertheless, the clinical translation of engineered probiotics faces substantial hurdles in manufacturing standardization, regulatory harmonization, and long-term biosafety assessment—challenges that are further discussed in the Safety and Challenges section.

### 5.2 Ecological remodeling: prospects of fecal microbiota transplantation as a disease-modifying therapy

FMT is emerging as a cutting-edge ecological therapy for intervening in the progression of T1D (132, 133). Currently, FMT is primarily administered via oral lyophilized capsules, colonoscopy, or enema. In T1D patients complicated with severe gastrointestinal lesions, repeated FMT, particularly in the form of oral capsules, has been proven effective in alleviating symptoms such as diarrhea and abdominal distension, as well as restoring normal stool consistency. However, the maintenance of therapeutic efficacy generally requires booster treatment every approximately five months (134). More promisingly, preliminary clinical studies have indicated that autologous lyophilized fecal microbiota capsules (a-LFMCs) may have the potential to stabilize C-peptide levels and slow the decline of β-cell function, thereby providing early evidence for the direct intervention of FMT in the core pathogenesis of T1D (135). However, the clinical application of FMT is tempered by unresolved issues regarding donor screening standardization, informed consent in pediatric populations, and the lack of harmonized regulatory oversight across jurisdictions. For FMT, the blind transfer of complex consortia risks the inadvertent introduction of pathobionts or oncogenic viral elements, which could exacerbate the pre-existing autoimmune dysbiosis rather than rectify it.

An in-depth understanding of the mechanisms underlying FMT efficacy is driving the evolution of related technologies toward greater precision and safety. Studies have revealed that the therapeutic effects of FMT are partially attributed to key metabolites such as SCFAs produced by the transplanted microbiota (136). Based on this, emerging strategies are no longer confined to intact fecal transplantation. On the one hand, autologous fecal microbiota transplantation enhances safety by eliminating the need for allogeneic donors (135). On the other hand, research is underway to explore the direct delivery of synthetic microbial consortia or specific metabolites (e.g., SCFA capsules) as alternatives to traditional FMT. These approaches can be combined with advanced immunomodulatory technologies such as CAR-Treg therapy to achieve precise intervention in autoimmune responses under the guidance of multi-omics data (27, 137).

Although existing clinical evidence has demonstrated that FMT can improve glycemic control and insulin resistance in some patients (138), current evidence supporting FMT as a disease-modifying therapy in T1D remains extremely limited. Large-scale randomized controlled trials are still required to confirm its ability to alter the natural course of T1D (139).

### 5.3 Targeted delivery: technological prospects for ensuring efficient colonization and therapeutic efficacy of probiotics

Oral probiotic therapy for T1D is constrained not only by strain-specific efficacy but also by the limited survival, unpredictable intestinal retention, and inefficient delivery of viable bacteria to the sites where microbiota–immune interactions relevant to T1D occur (140). Advances in delivery technologies—such as polysaccharide hydrogels, nanoparticles, and microencapsulation—have effectively addressed the challenges of probiotic survival and gut localization, but it’s still in the preclinical trial stage (141, 142). These technologies enable probiotics to stably express therapeutic proteins or antigens in the intestine, thereby achieving precise intervention in autoimmune disease through the induction of Treg, modulation of SCFAs, and restoration of the intestinal barrier function. However, the existing research predominantly addresses targeted delivery therapy for Inflammatory bowel disease (IBD), whereas the focus on T1D therapy is relatively minor. This section reviews the latest literature on targeted delivery of probiotics to enhance the treatment of IBD. The aim is to provide guidance and insights to inform future targeted probiotic strategies for the prevention and treatment of T1D.

A growing body of research has focused on the use of nanomaterials and nanotechnology for probiotic delivery. Nanoencapsulation techniques entrap probiotics within nanoshell matrices, for instance, through single-cell encapsulation with nanocoatings (143, 144).

Currently, cationic polymers are commonly used to fabricate protective nanocoatings (145). As a typical cationic polymer, chitosan (CS) can be deposited onto the negatively charged surface of bacteria (e.g., alginate-coated *E. coli Nissle 1917*) via electrostatic attraction, forming a protective nanocoating that markedly enhances the stress resistance of probiotics under simulated GI conditions. (**Figure 6D**), (148, 149) Bilayer nanocoating B.B.@PDA@CeO_2_ was prepared by PDA biointerface self-assembly and CeO_2_ NPs electrostatic adsorption. Direct modification on single-cell probiotics with this coating notably increased the survival of *Bifidobacterium bifidum (B.B.)* under simulated gastric acid conditions. (**Figures 6A, B**), (146) Versatile inulin/trans-ferulic acid/silk sericin nanoparticles-nourished probiotics effectively prolong the intestinal residence time of probiotics. (**Figure 6C**), (147).

**Figure 6:**
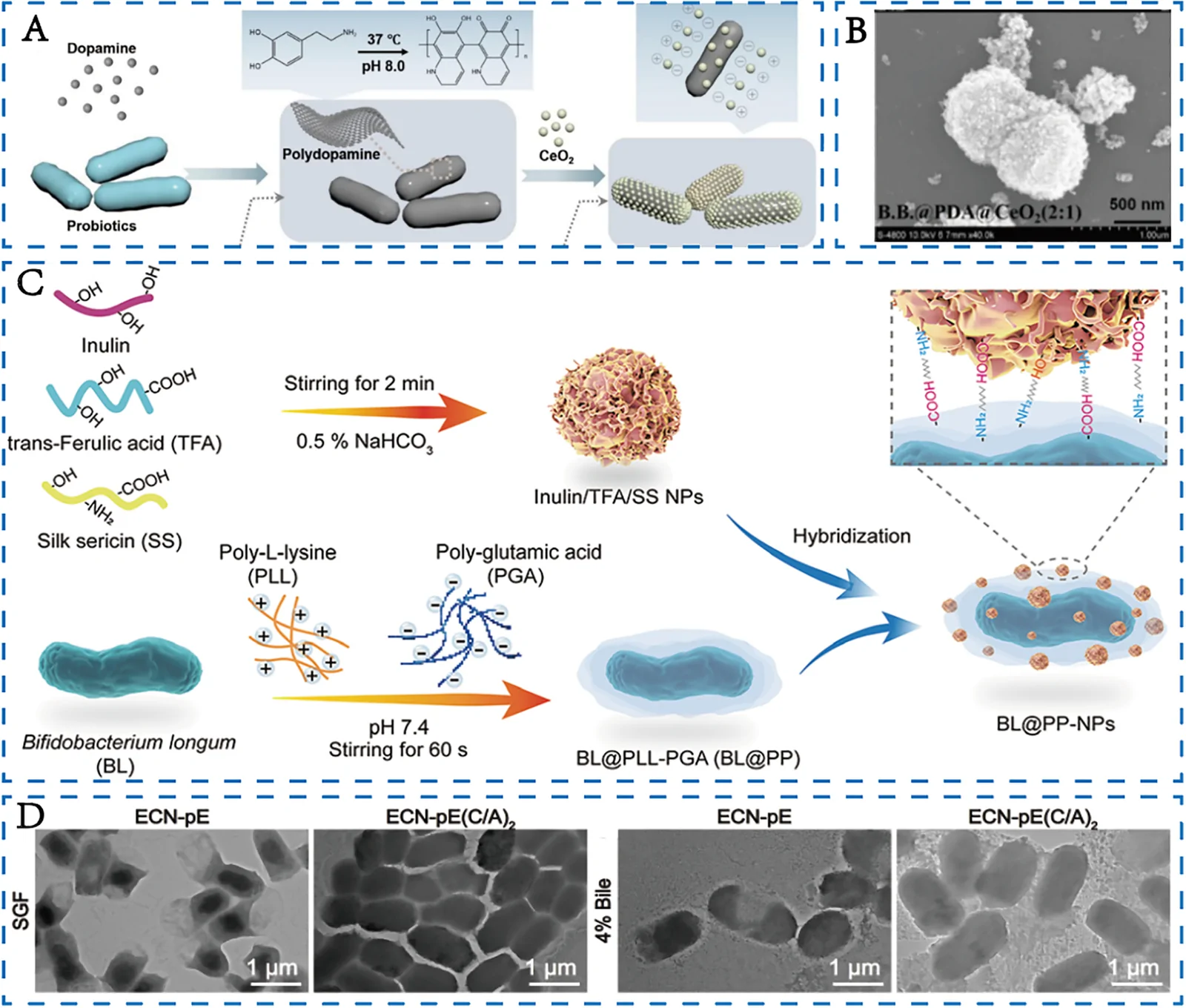
**(A)** Schematic illustration of the formation of B.B.@PDA@CeO2. **(B)** SEM and TEM images of B.B.@PDA@CeO2. Reproduced with permission (146). Copyright 2025, Elsevier. **(C)** Schematic diagram of the preparation of BL@PP- NPs. Reproduced with permission (147). Copyright 2025, Elsevier. **(D)** Representative TEM images of ECN-pE and ECN-pE(C/A)2 after exposure to SGF or 4% bile salt at 37 °C for 2 h from two independent samples. Scale bar: 1 μm. Reproduced with permission (148). Copyright 2025, Springer.

It has been documented that composite hydrogels combining polysaccharides and proteins take advantage of the complementary functions of different polymers, while also boosting stomach acid protection, structural stability, mucosal adhesion, and stimulus-responsive release. To cope with the complex digestive tract environment, multilayer coating and composite hydrogel technologies have been extensively developed. Layer-by-layer assembly of materials such as chitosan, (**Figure 7A**) (150) sodium alginate, and liposomes, a dual-targeting property to MnCe@LR/AMs, (**Figure 7B**) (151) or the construction of double-network structures cross-linked by disulfide bonds and ionic interactions (**Figure 7C**), (152) can significantly improve the survival rate of probiotics under gastric acid conditions and their adhesion to the intestinal mucosa. Double-layer alginate microcapsules (DAM) with resistant starch nanoparticles (RSNP) as the outer shell have been shown to increase the survival rate of *Lactobacillus plantarum* to 52% under gastric acid (pH<2) and promote the production of SCFAs, (**Figure 7D**) (153) offering a new perspective for T1D treatment.

**Figure 7:**
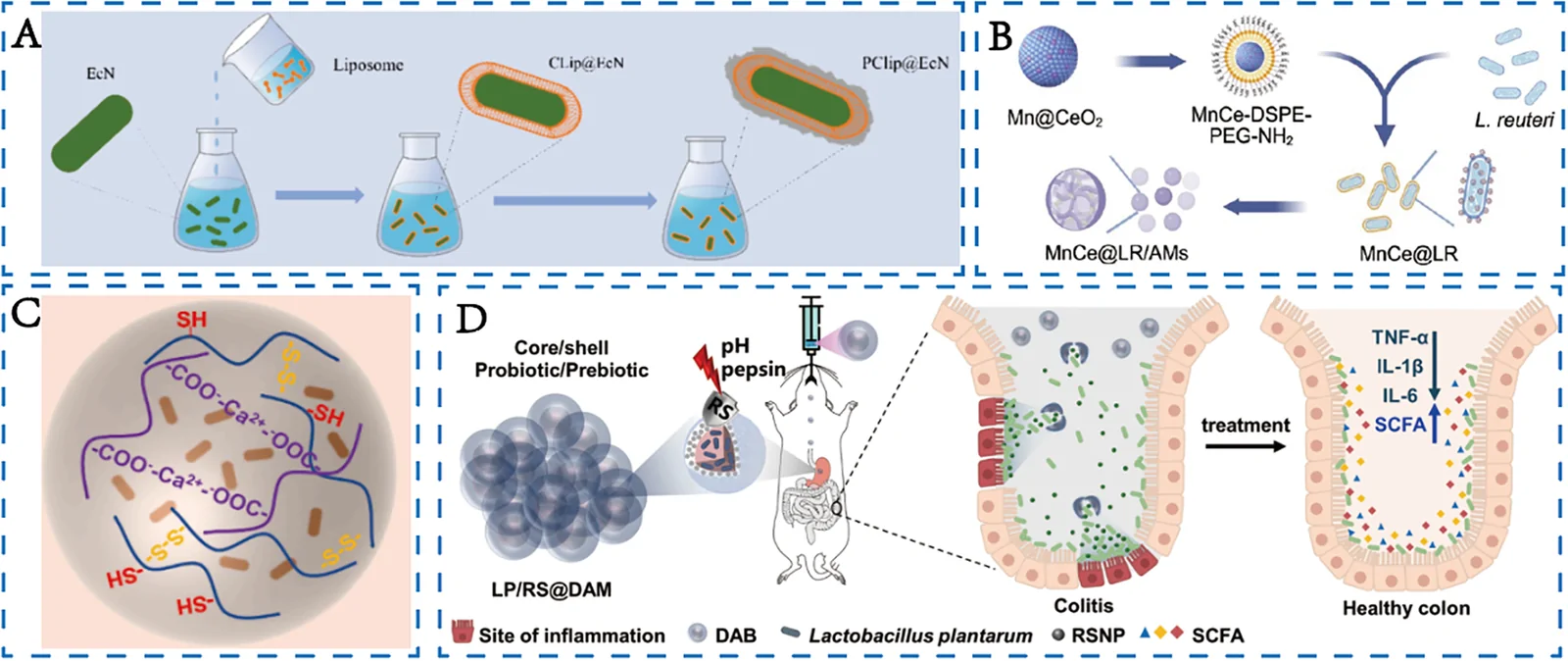
**(A)** Diagram illustrates the PCLip@EcN preparation process. Reproduced with permission (150). Copyright 2024, Elsevier. **(B)** MnCe@LR/AMs, synthesized from Mn@CeO 2 nanozymes. Reproduced with permission (151). Copyright 2025, ACS Publications. **(C)** Design of mucoadhesive and intestinal-targeted SOGG/SA microspheres loaded with LGG. Reproduced with permission (152). Copyright 2025, Elsevier. **(D)** Schematic diagram of double-layer alginate microcapsules (LP/RS@DAM) loaded with LP/RSNP (core/shell) for colitis treatment. Reproduced with permission (153). Copyright 2025, Elsevier.

Furthermore, functional modification of biomaterials enables targeted therapy of diseases. For example, hyaluronic acid is used to target inflammatory sites, while tannic acid (TA) modification or grafting of gold nanozymes can scavenge excessive reactive oxygen species (ROS), thereby synergizing with probiotics to regulate local immune responses and oxidative stress levels (**Figures 8A–C**) (154–156). In addition, biomimetic and dynamic chemical protection strategies have demonstrated great potential. For instance, yeast cell wall microparticles are employed to mimic natural carriers for colon-targeted delivery, and dynamically covalent coatings based on Schiff bases are designed to provide full-course protection in the GI tract while enabling site-specific release of probiotics at inflammatory loci for immune modulation (**Figures 8D, E**), (157, 158).

**Figure 8:**
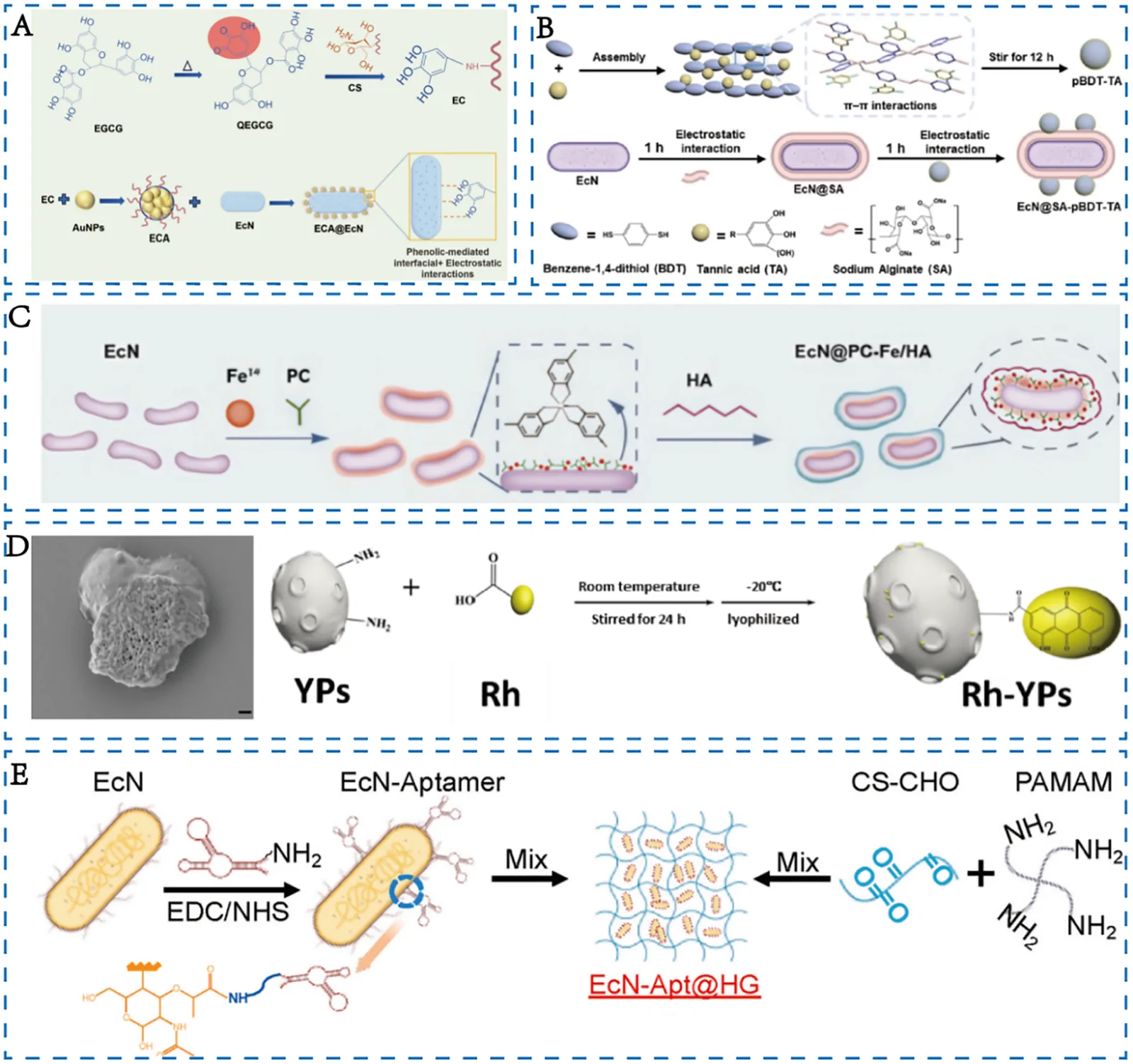
**(A)** ECA@EcNwasconstructedthroughphenolic-metal coordination-mediated interfacial and electrostatic interactions. Reproduced with permission (154). Copyright 2025, Advanced Science. **(B)** Schematic Representation of pBDT-TA Nanoparticles Prepared by π−π Interaction with BDT and TA. Escherichia coli Nissle 1917 (EcN) with the Sodium Alginate (SA) Layer and Coating pBDT-TA to the Surface of SA via Electrostatic Interaction. Reproduced with permission (155). Copyright 2024, ACS Publications. **(C)** Demonstration of a synthetic procedure for EcN@PC-Fe/HA with PC, Fe III, and HMW-HA by layer-by-layer coating strategy. Reproduced with permission (156). Copyright 2025, Wiley. **(D)** SEM image of YPs and Rh-YPs, and schematic illustration of probable combination form about Rh and YPs. Scale bar: 1 μm. Reproduced with permission (157). Copyright 2025, Advanced Science. **(E)** Preparation of EcN-Apt@HG. Reproduced with permission (158). Copyright 2025, Elsevier.

The design of these delivery systems not only improves the survival rate and targeting ability of probiotics but also expands their application potential in disease treatment, thus laying a crucial technical foundation for the intervention of autoimmune diseases such as T1D via the microbiota-immune axis.

Delivery concepts developed specifically for diabetes provide a more direct basis for adapting probiotic delivery to T1D than extrapolation from intestinal inflammatory diseases alone. Oral insulin delivery systems with distinct targeting mechanisms—such as arabinoxylan microspheres relying on colon-specific targeting and pH-responsive Chlorella-sodium alginate hydrogels—can effectively protect insulin from degradation in the upper gastrointestinal tract, achieve site-specific release in the intestine, exert significant hypoglycemic effects in diabetic models, and synergistically improve insulin sensitivity and metabolic homeostasis by modulating the gut microbiota and their metabolites (159, 160). Ursodeoxycholic acid (UDCA) microcapsules have been developed as a targeted delivery system that effectively lowers blood glucose and inflammation levels through precise regulation of cholic acid and lithocholic acid concentrations, achieving direct protection of pancreatic β-cells (161). Despite significant advancements in nano-delivery systems for insulin or metabolite formulations, their application in the direct, *in-situ* delivery of live probiotics to precisely modulate the gut-immune-pancreas axis remains an underexplored challenge in the field of T1D treatment.

Most advanced probiotic delivery systems remain at the preclinical stage, and direct evidence demonstrating improved disease modification in T1D is still limited; therefore, their value in T1D should currently be regarded as a promising translational strategy rather than an established therapeutic approach.

### 5.4 Personalized strategy: towards a new era of individualized probiotic therapy

The gut microbiota is closely associated with the pathogenesis of T1D, making personalized probiotic intervention a research hotspot. The gut microbiota is influenced by multiple factors such as host genetic background, dietary structure, antibiotic use history, and age (162–165). The unpredictable colonization efficacy of probiotics prevents traditional probiotic formulations from being widely applied to all individuals. The high inter-individual variability of the gut microbiota renders the conventional “one-size-fits-all” probiotic strategy ineffective, resulting in unpredictable and irreproducible intervention outcomes.

However, this predicament precisely points out the direction for breakthroughs in emerging technologies. In the future, precision therapies integrating artificial intelligence (AI) and microbiome analysis will become the key to addressing this challenge. Personalized probiotic therapies that combine AI and microbiome analysis can optimize strain selection and dosage to achieve precise regulation (23). Through deep metagenomic sequencing and multi-omics data integration, AI models can systematically decipher the unique microbiota-immune-metabolism interaction network of each individual, and accurately identify the core targets of microbiota functional deficiency or disorder (166).

On this basis, machine learning algorithms can perform high-throughput screening and matching of the most suitable probiotic strains or consortia, dynamically optimize the dosage and timing of administration, and even predict the synergistic effects between probiotics and the host’s diet (167–169). This integrated closed-loop strategy of “diagnosis-decision-intervention” is expected to upgrade probiotic intervention from the current extensive empirical supplementation to a quantifiable, predictable, and truly personalized precision regulation approach, thereby opening up a brand-new avenue for the immune intervention of T1D.

Despite the theoretical appeal of the above framework, its clinical implementation faces multiple barriers. First, at the data level, there is a lack of large-scale, standardized, longitudinally followed multi-omics datasets for training and validating AI models; most available studies are cross-sectional and fail to capture the dynamic changes of the microbiota. Second, at the algorithm level, the limited interpretability of machine learning models hinders clinical trust and adoption of AI-recommended strain combinations. Third, at the clinical level, high costs and technical complexity limit widespread implementation; moreover, the relatively long onset time of microbiota-directed interventions and substantial inter-individual variability complicate efficacy assessment.

## 6 Safety and challenges

In human clinical trials, most commonly used probiotic strains have generally been well tolerated, and available evidence has not demonstrated a consistent increase in adverse events compared with control groups; however, adverse-event reporting remains incomplete and heterogeneous across studies (170).

Not all probiotic strains are suitable for the treatment of inflammatory diseases or cancer; some strains may induce adverse reactions, including abdominal bloating and diarrhea (171). Moreover, probiotic therapy is not applicable to every individual. For instance, cases of bacteremia associated with probiotic treatment have been documented in particularly vulnerable populations, such as very young children and children with compromised immune function (172, 173). The intestinal barrier dysfunction commonly observed in T1D patients may increase the risk of bacterial translocation. For pediatric T1D patients and other potentially vulnerable populations, greater emphasis should be placed on patient screening and clinical monitoring when considering probiotic interventions. In addition, the potential risks of long-term or excessive probiotic use remain unclear, including microbial imbalance and excessive immune activation (174). So clinical trials should include baseline risk assessments, clear exclusion criteria, necessary microbiological monitoring, and systematic reporting of adverse events.

Safety evaluation of engineered probiotics mainly focuses on three aspects: genetic stability, risk of horizontal gene transfer, and host immune responses. Multiple studies have demonstrated that GRAS (Generally Recognized as Safe) -grade strains (e.g., *L. lactis, EcN*) do not trigger systemic inflammation or yield positive blood culture results after high-dose oral administration (175). The co-stimulatory molecules (CD80/86) knocked out by CRISPR-Cas9 maintain low expression levels *in vivo*, without inducing immune deficiency (130). However, the transition of engineered probiotics into a mainstream treatment option for T1D faces significant barriers that far exceed those related to their biological efficacy alone (176). Firstly, the ethical considerations associated with the use of genetically modified microorganisms (GMOs) in human subjects necessitate rigorous oversight, transparent patient communication, and particularly cautious evaluation when targeting pediatric and adolescent populations (177). Secondly, unlike traditional small-molecule drugs, engineered probiotics require manufacturing processes compliant with Good Manufacturing Practice (GMP) standards to ensure the consistency of bacterial strain engineering, genetic stability, scalable production capacity, and sterility between batches. Preclinical toxicology studies and GLP (Good Laboratory Practice) -GMP production processes have achieved batch-to-batch consistency, laying a solid foundation for subsequent Phase III clinical trials (178). Finally, the long-term biosafety of engineered bacterial strains has not yet been fully elucidated. Outstanding questions remain, including the potential for horizontal gene transfer among indigenous gut microbiota, the unintended ecological consequences arising from persistent colonization, and the immunogenicity of recombinant proteins or genetic circuits in long-term therapeutic applications.

In addition, several challenges still hinder the widespread application of probiotics in the treatment of T1D. The development of novel delivery technologies has emerged as an emerging research direction. The construction of probiotic delivery systems relies on the synergistic interaction of multiple materials, which form composite carriers through covalent or non-covalent bonding. However, the selection of bonding strength is crucial: excessively strong bonding may lead to delayed release of probiotics at target sites, whereas insufficient bonding strength can cause premature release of probiotics before they reach the intended location (179). Moreover, certain synthetic components—such as surfactants and polymers used in oral delivery systems—may pose biotoxicity risks, potentially disrupting gut microbiota homeostasis or triggering gastrointestinal inflammatory responses (180, 181). Therefore, an ideal delivery material should integrate targeting capabilities (e.g., ROS responsiveness or CD44 receptor-binding affinity) with biosafety properties including biocompatibility, biodegradability, protective efficacy and non-toxicity.

Currently, the preparation of probiotic delivery systems involves complex procedures, including probiotic cultivation, material synthesis, carrier fabrication and drug loading. Laboratory research often relies heavily on manual operations, making it difficult to achieve high-level automation and standardization. This results in significant variations across different preparation batches. Errors during manual manipulation can compromise probiotic viability, drug loading efficiency and the overall performance of the delivery system, thereby hindering the guarantee of consistent product quality and functionality. This challenge is particularly pronounced in clinical applications requiring high reproducibility and precise controllability, such as T1D treatment, and has become a major bottleneck for technology translation.

The quality and potency of probiotics also directly affect their therapeutic efficacy. The lack of standardized probiotic strains, dosages and treatment regimens impairs the comparability of research outcomes. It is imperative to verify the safety and efficacy of probiotic therapy through multicenter, large-sample clinical trials, and establish unified standards for probiotic strain selection, dosage determination and treatment protocols to enhance the comparability of results.

According to the analysis above, the functions of probiotics are associated with inherent limitations. The therapeutic mechanisms of probiotics in T1D have not been fully elucidated, which restricts their precise clinical application (182). While certain strains promote Treg differentiation and immune tolerance, others may paradoxically exacerbate autoimmunity. For instance, specific Lactobacillus strains have been shown to enhance Th17 responses via the AhR pathway, potentially amplifying the autoimmune attack on pancreatic β-cells (50). More importantly, probiotics alone may not address complex inflammation or immune-related disorders and may need to be combined with other therapeutic modalities. In-depth investigation into the mechanisms of probiotics, particularly their anti-inflammatory and immunomodulatory effects in T1D, is crucial for providing a theoretical basis for precision medicine.

Given the inter-individual differences in gut microbiota composition and inflammatory microenvironment among patients with T1D, future research should leverage technologies such as metagenomics to analyze the gut microbiota of individual patients and formulate personalized probiotic regimens. Combining probiotics with small-molecule drugs or immune checkpoint inhibitors may yield synergistic therapeutic effects. Implementing multi-layered gut microecology modulation strategies, which integrate dietary interventions, prebiotics and postbiotics, represents a promising direction.

## 7 Conclusion

The potential of probiotics in the prevention and adjuvant treatment of T1D has attracted increasing attention, which has sparked a surge of interest in the development of sophisticated targeted delivery systems and AI-assisted personalized therapies. Although the therapeutic efficacy of probiotics against T1D has been validated in numerous animal experiments, it has not achieved the expected outcomes in clinical trials. It must be emphasized that, probiotics should be considered an adjunctive—rather than replacement—therapy for T1D until more robust clinical data become available. Strategies such as hydrogel microcapsules, nanofibers, and even single-cell “nano-armor” have significantly improved the survival rate, retention time, and colonization efficiency of probiotics in the gastrointestinal tract. The exploration of engineered probiotics also offers new insights for therapeutic development. However, current research still faces several challenges. Most studies focus on single strains or specific mixed formulations, lacking systematic dose-response curves and long-term safety evaluations. Additionally, differences in immune backgrounds among various experimental models limit the direct extrapolation of research results. Future research should conduct large-scale animal and clinical trials to compare the efficacy of different microbial combinations and identify the optimal intervention window. Moreover, interdisciplinary approaches integrating artificial intelligence, metagenomics, metabolomics, and materials science are needed to clarify the molecular details of microbe-host interactions, and to develop efficient personalized probiotic therapies, thereby laying a more solid foundation for subsequent clinical translation.
